# An individual with Sarmatian-related ancestry in Roman Britain

**DOI:** 10.1016/j.cub.2023.11.049

**Published:** 2023-12-19

**Authors:** Marina Silva, Thomas Booth, Joanna Moore, Kyriaki Anastasiadou, Don Walker, Alexandre Gilardet, Christopher Barrington, Monica Kelly, Mia Williams, Michael Henderson, Alex Smith, David Bowsher, Janet Montgomery, Pontus Skoglund

**Affiliations:** 1 Ancient Genomics Laboratory, The Francis Crick Institute, 1 Midland Road, London NW1 1AT, UK; 2 Department of Archaeology, Durham University, Lower Mountjoy, South Rd, DH1 3LE, Durham, United Kingdom; 3 Museum of London Archaeology (MOLA), Mortimer Wheeler House, 46 Eagle Wharf Road, London N1 7ED, UK; 4 Bioinformatics and Biostatistics, The Francis Crick Institute, 1 Midland Road, London NW1 1AT, UK; 5 Headland Archaeology, 13 Jane Street, Edinburgh EH6 5HE, UK

## Abstract

In the second century CE the Roman Empire had increasing contact with Sarmatians, nomadic Iranian speakers occupying an area stretching from the Pontic-Caspian steppe to the Carpathian mountains, both in the Caucasus and in the Danubian borders of the empire.^1–3^ In 175 CE, following their defeat in the Marcomannic Wars, emperor Marcus Aurelius drafted Sarmatian cavalry into Roman legions and deployed 5,500 Sarmatian soldiers to Britain, as recorded by contemporary historian Cassius Dio.^4,5^ Little is known about where the Sarmatian cavalry were stationed, and no individuals connected with this historically attested event have been identified to date, leaving its impact on Britain largely unknown. Here we document Caucasus- and Sarmatian-related ancestry in the whole genome of a Roman-period individual (126–228 calibrated [cal.] CE)—an outlier without traceable ancestry related to local populations in Britain—recovered from a farmstead site in present-day Cambridgeshire, UK. Stable isotopes support a life history of mobility during childhood. Although several scenarios are possible, the historical deployment of Sarmatians to Britain provides a parsimonious explanation for this individual’s extraordinary life history. Regardless of the factors behind his migrations, these results highlight how long-range mobility facilitated by the Roman Empire impacted provincial locations outside of urban centers.

## Graphical abstract

**Graphical abstract:**
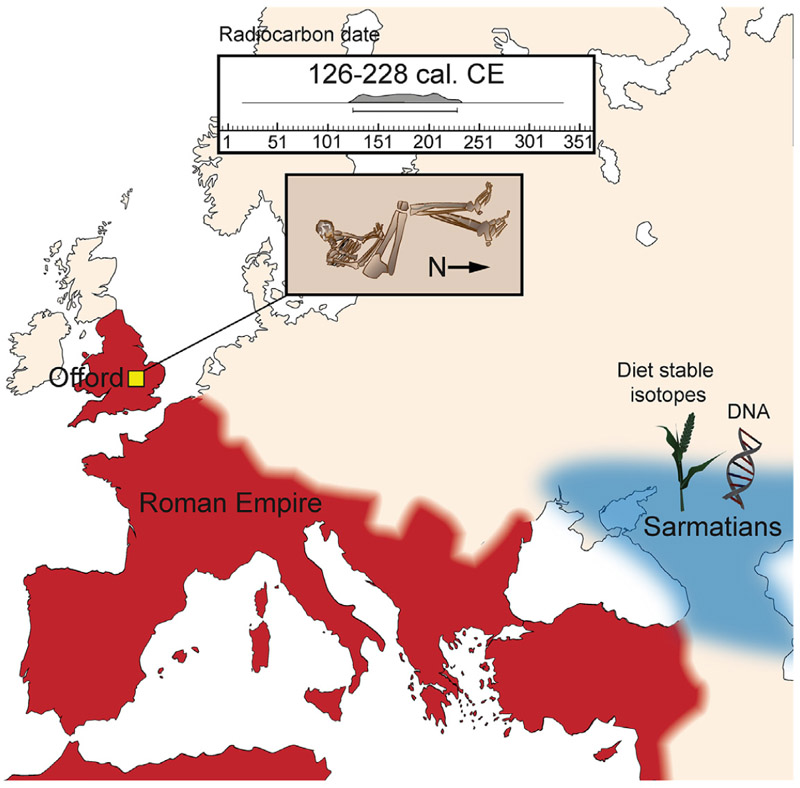

## Results

### An ancestry outlier in rural Roman Cambridgeshire

**Figure 1:**
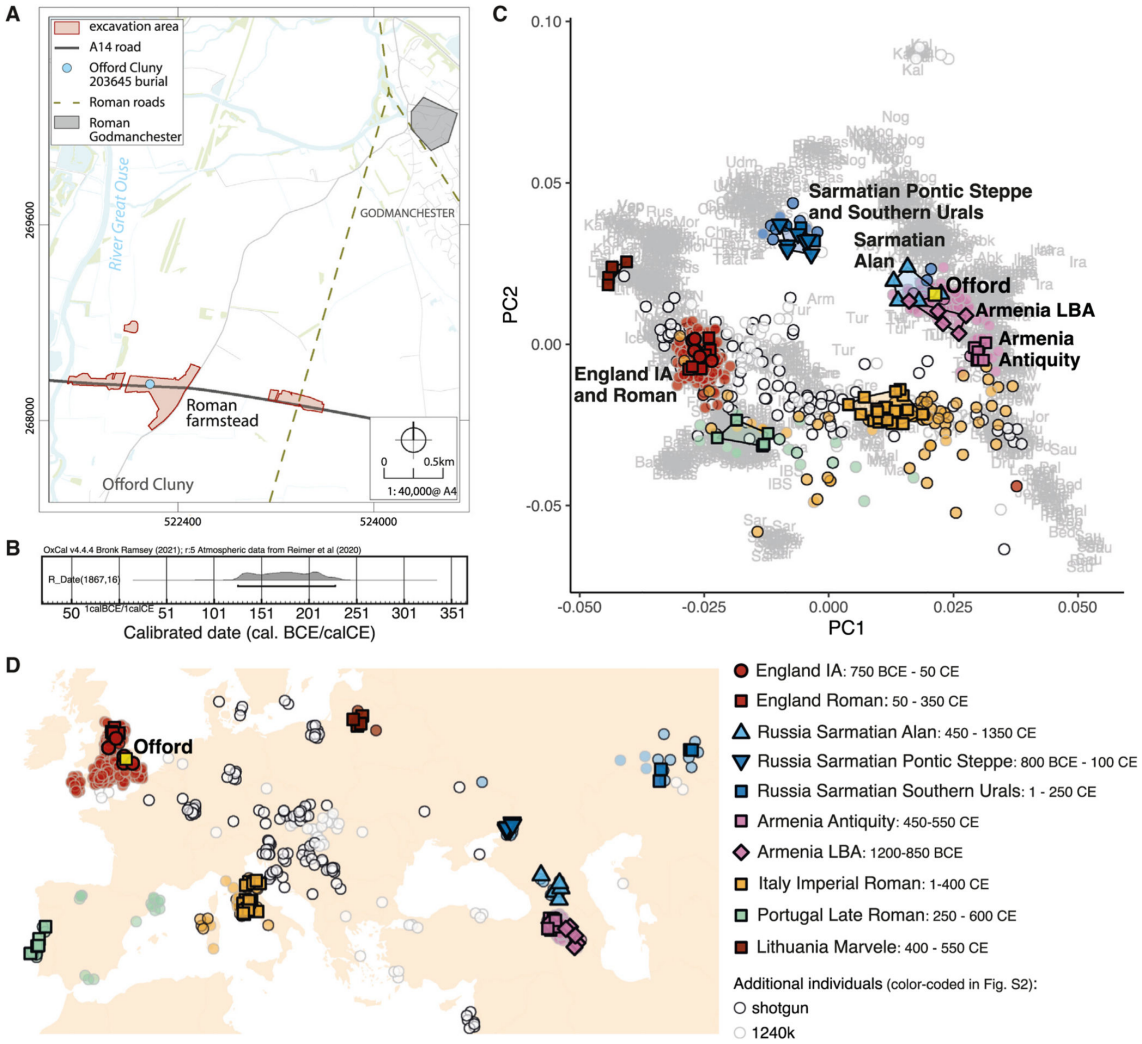
Ancestry outlier Offord Cluny 203645 (A) Map of the site, showing the excavation area and the location of burial relative to Roman roads and Roman Godmanchester. Burial shown in Figure S1; for sequencing metrics and uniparental haplogroups see Data S1. (B) Calibrated radiocarbon date (126–228 cal. CE) of Offord Cluny 203645’s second right maxillary molar using OxCal v4.4^7^ and IntCal20^8^ (1,867 ± 16 years before present [BP], SUERC-105720 [GU61561]). (C) Principal component analysis (PCA) showing Offord Cluny 203645 (yellow square) and other previously published ancient individuals projected onto PCs defined by 1,388 present-day western Eurasian individuals from the Affymetrix Human Origins (HO) ~600k SNP panel. Individuals included in the populations used as sources in the *qpWave/qpAdm* models are highlighted, with additional individuals from the same regions colored according to geography (shown in D). For a detailed caption of all projected ancient individuals see Figure S2A; *f*_4_-statistics shown in Figure S3. Present-day individuals are indicated by the first 3 letters of their population label, as reported in Data S2E. (D) Map of ancient individuals included in PCA (with added jitter) and approximate calibrated dates of populations used as references in proximal models tested with *qpWave/qpAdm* framework (shown in Figure 2). Offord Cluny 203645 is represented by a yellow square. Data points colored according to geography and data type (whole-genome shotgun sequencing or “1240k” SNP capture), additional individuals are color-coded in Figure S2A. See also Data S1 and S2.

Human remains were recovered from an isolated burial during excavations near the village of Offord Cluny led by MHI (Museum of London Archaeology [MOLA] Headland Infrastructure) in advance of the National Highways A14 road development in Cambridgeshire, England (Figures 1A and S1A). We generated a ~5.4× whole genome from the cochlea portion of the temporal bone of the Offord Cluny skeleton (Sk 203645, Burial 20.507, C10271), using single-stranded DNA library preparation (STAR Methods; Data S1A). A tooth was directly radiocarbon dated to 126–228 cal. CE (95% confidence, SUERC-105720), in the early-mid Roman period (Figure 1B). The skeleton was only moderately well preserved macroscopically: although osteological analysis of the remains suggested the individual was 18–25 years old, it was not possible to produce a sex estimate. Although there were some osteological indications of minor trauma in the past, there was nothing to suggest a cause of death. Assessment of karyotypic sex^6^ using the sequenced genome established that the remains belonged to a male individual (XY).

In a principal-component analysis (PCA), Offord Cluny 203645 is differentiated from all other sampled Roman individuals from Britain, excavated from a Roman cemetery at Driffield Terrace, in the present-day city of York, northeast England (*England_Roman*, excluding a previously described outlier with ancestry related to Near Eastern populations).^9^ Instead, Offord Cluny 203645 is most similar to present-day individuals from Anatolia and the Caucasus (Figures 1C and S2A). Specifically, he shows affinities to Late Bronze Age individuals from Armenia (*Armenia_LBA*) and individuals recovered from Alan-associated contexts in the North Caucasus (*Russia_Sarmatian_Alan*, dating to 450–1350 CE,^10^ generally considered as part of the Sarmatian confederation^11^), but not with individuals from Armenia who post-date the Bronze Age (here defined as *Armenia_Antiquity*^12^) (Figure 1D).

Similarly, direct statistical tests in the form of *f*_4_-statistics consistently show that the genetic ancestry of the Offord Cluny individual was different from the ancestry of Romano-British individuals from Driffield Terrace, and he instead shared genetic affinities with ancient populations from the Caucasus and Pontic-Caspian region (Figure S3 and Data S2A).

Analysis of the Y chromosome and mitochondrial DNA (mtDNA) of Offord Cluny 203645, tracing paternal and maternal lineages, respectively, also point to ancestry from outside of western Europe, in particular his paternal lineage: R1b-Y13369 (a sub-branch of R1b1a1b1b/R1b-Z2103) (Data S1B). This lineage has been previously identified in skeletal remains ranging from the Late Bronze Age to the Urartian period recovered from present-day Armenia,^13^ whereas its present-day phylogeny is dominated by samples from the Caucasus, Anatolia, and Near East (Yfull tree v.11.01.00). Offord Cluny carried mtDNA haplogroup K1a (Data S1C), found in Pre-Pottery Neolithic Anatolia and the Levant, and in Europe since the Neolithic.^14,15^ Although subclades of haplogroup K1a, found at frequencies of ~5% across all regions in the UK Biobank dataset,^16^ have been previously identified in ancient individuals from Britain ranging from the Neolithic to the early Medieval period, these all belong to different sublineages than the one observed in Offord Cluny.^17–21^

### Relationship to Caucasus and Sarmatian groups

**Figure 2:**
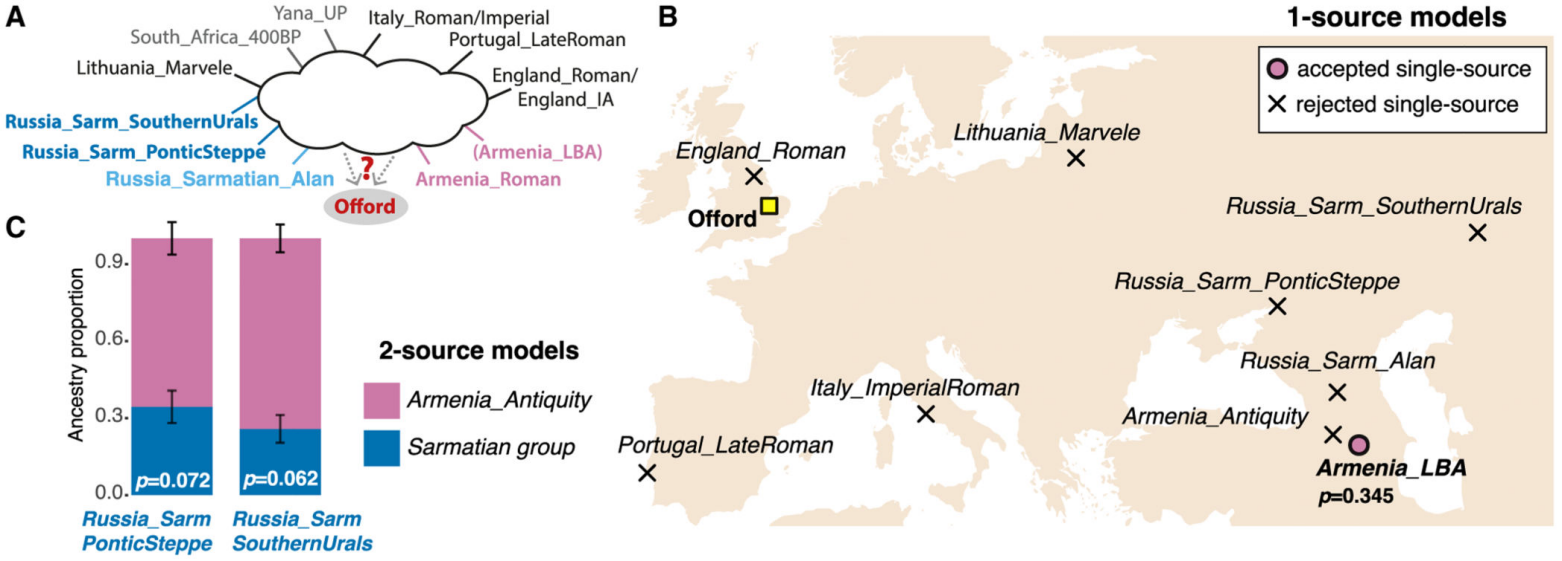
Ancestry modeling for Offord Cluny 203645 (A) Rotating models tested using *qpWave/qpAdm* framework. *Armenia_LBA* was excluded when testing 2-source models (shown in C). Models shown in (B) and (C) used *England_Roman*; models using *England_IA* are shown in Data S2B and S2C. (B) Location of populations included in the *qpWave* model (*South_Africa_400BP* and *Yana_UP* not shown) and p value for the single-source model accepted (p value > 0.05); additional tested models shown in Data S2B. Location of the A14 site where Offord Cluny 203645 was found is indicated by a yellow square. (C) Accepted 2-source *qpAdm* model for individual Offord Cluny 203645 when rotating through temporally proximal source (p > 0.05); all tested models shown in Data S2C. Models using distal sources shown in Figure S2. See also Data S2.

With the PCA having established the broad affinities of Offord Cluny 203645, we moved on to testing explicit ancestry models with the *qpWave/qpAdm* framework. This approach allows us to test ancestry models and statistically reject those that do not fit the data. Our goal was to find models that uniquely fit the ancestry of the Offord Cluny 203645 individual—i.e., where all other models of similar complexity (number of distinguishable ancestries) are rejected—with the caveat that we are limited to the data available in the literature from approximately contemporaneous periods from other regions. We first tested different single-source *qpWave* models rotating through different populations (STAR Methods), with a focus primarily on populations from the Caucasus and the Pontic-Caspian steppe, in addition to other populations from south and northern Europe (Figure 2A). The only accepted single source is *Armenia_LBA* (p values = 0.345 and 0.560), whereas *Armenia_Antiquity*, Sarmatian groups, and populations from Britain (*England_Roman* or *England_IA*) are rejected as single sources (Figure 2B and Data S2B).

However, *Armenia_LBA* dates to ~1200–850 BCE and thus predates Offord Cluny 203645 by up to approximately one millennium. Recent studies revealed ancestry changes in Armenia during the first millennium BCE, which resulted in different ancestry patterns in the region by the time of Offord Cluny 203645.^12,13^ Therefore, *Armenia_LBA* is likely not a good representative of the ancestry observed in the Caucasus in the first millennium CE (Figure 1C). With this in mind, we tested additional models excluding *Armenia_LBA* (Figure 2C), which were consistent with Offord Cluny 203645 carrying ~24%–34% of his ancestry from a source close to Sarmatian groups from the Pontic-Caspian region (either *Russia_Sarmatian_PonticSteppe* or *Russia_Sarmatian_SouthernUrals*), in addition to ancestry from a source most similar to *Armenia_Antiquity* (p values ranging from 0.062 to 0.124, and standard errors (SEs) varying from 5% to 6%, depending on the model; Data S2C). We note that a third similar model, with *Russia_Sarmatian_Alan* and *Armenia_Antiquity* as sources, is just under the threshold of significance (p value ~0.030, Data S2C). Overall, our results suggest that there may have been substantial diversity among groups identified as Sarmatians, some of which could have had ancestry that in our data is most closely represented by *Armenia_Antiquity*.

### Stable isotopes support long-distance mobility

**Figure 3:**
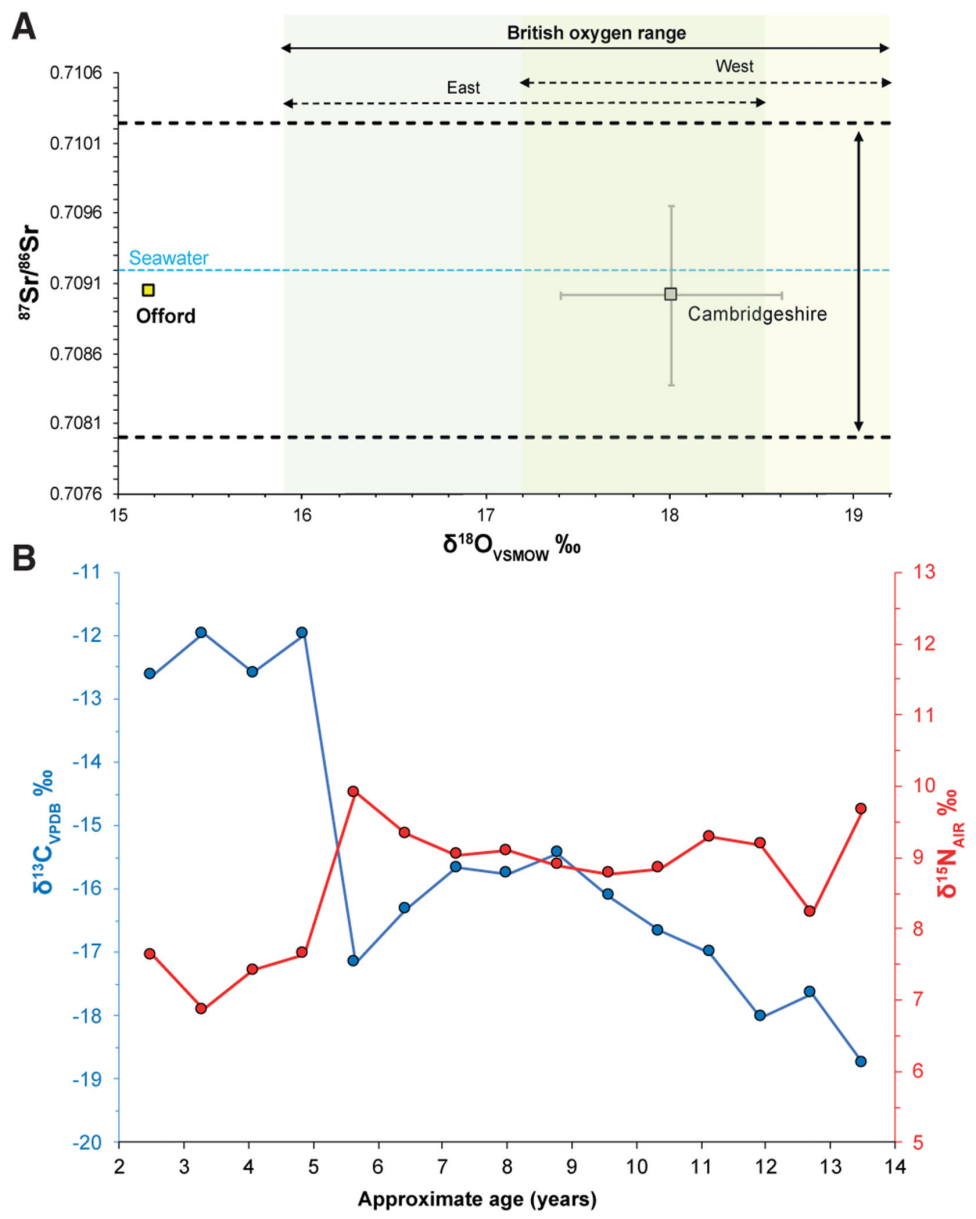
Stable isotope analyses (A) Offord Cluny 203645 human tooth enamel strontium (Sr) and oxygen (O) isotope data (Data S3A) alongside mean (± 1 SD) regional comparative data.^28,29^ The horizontal dotted lines represent the bioavailable Sr isotope range for Cambridgeshire.^23^ The shaded green and yellow boxes represent the 2 SD O isotope range expected for east and west Britain, respectively.^24^ Analytical error for O is 0.28‰, 1 SD, and Sr is within the symbol. (B) Diet changes in the first 14 years of Offord Cluny 203645’s life as indicated by incremental dentine δ^13^C and δ^15^N data (second right mandibular molar, M2) plotted against approximate age in years (see also Data S3B).

The results of the carbon (C), nitrogen (N), oxygen (O), and strontium (Sr) isotope analyses are presented in Figure 3. The ^87^Sr/^86^Sr value from Offord Cluny 203645’s second mandibular molar (reflecting the first 5 to 6 years of his childhood^22^) was 0.709037 ± 0.000012 (2 SE), and strontium concentration from the same tooth was 104.2 parts per million (ppm), both of which are within the range expected for Britain^23,24^ (Figure 3A and Data S3A). However, this is a common ^87^Sr/^86^Sr ratio that can be produced by a wide range of geological terrains, and humans with similar values can be found in a variety of places. On the other hand, δ^18^O values were lower than what would be expected if he had spent the first years of his childhood in Britain (Figure 3A and Data S3A) and are instead indicative of regions with a colder or more continental climate, being consistent with levels of precipitation recorded today in regions at high altitude.^25^ Similar combinations of Sr and O isotope ratios have been observed in Roman-period populations in continental Europe.^26,27^

Offord Cluny 203645 had high δ^13^C values combined with low δ^15^N values, indicating a childhood diet rich in non-native C_4_ crops with little input from marine resources. Incremental dentine analysis (Figure 3B and Data S3B) revealed that his diet underwent a substantial change around the age of 5 years, when δ^13^C values drop from ~−12‰ to ~ −16‰, reflecting a clear shift from eating predominantly C_4_ plant protein to eating a mixed C_3_/C_4_ diet with a possible increase in meat protein indicated by a concomitant rise in δ^15^N. A second change in diet occurred after the age of 9, when the δ^13^C profile started falling, reaching ~ −19‰ around the age of 13, which is approaching an entirely C_3_ based diet. As there is no clear evidence of wide consumption of C_4_ crops during the Roman occupation of Britain (despite some sporadic findings of millet)^30^ and they were not common components of diet in western provinces of the Roman Empire, these two shifts in diet could represent a relocation around the age of 5 years old and again, after the age of 9 years old, which could reflect at least two periods of movement across Europe within the first ~14 years of his life. It is not possible to distinguish a gradual one-way transition in diet over several years of life from a fairly rapid change, due to increased overlapping in the orientation of the dentine incremental layers.^31,32^ Nevertheless, the gradual drop in δ^13^C values observed after the age of 9 could reflect either a sustained increased consumption of C_3_ crops over several years or possibly a multi-year migration, e.g., westward across Europe to Britain, through regions of gradually diminishing availability of C_4_ foods such as millet.

## Discussion

We have shown that the ancestry of Offord Cluny 203645 did not match that of the overall Romano-British population and that, instead, he shared genetic affinities with groups from the Caucasus and the Pontic-Caspian steppe. Complex patterns of ancestry in the Caucasus^12,13^ and sparse sampling in the region, particularly in the North Caucasus, covering the first four centuries CE hinder the identification of a single proximal source for his ancestry. Future sampling in western Eurasia—and specifically in the Pontic region and/or the North Caucasus—covering the first and second centuries CE will have the potential to help narrow down Offord Cluny 203645’s ancestry, possibly allowing the identification of a single temporally proximal source of ancestry.

Genetics alone provide little insight on mobility within the lifetime of one individual. Isotopic information is necessary for investigating lifetime mobility patterns. Taken together, the C, N, Sr, and O isotope analyses indicate that Offord Cluny 203645 spent the first 5 to 6 years of his childhood in a more eastern and arid continental location. This could include regions within the empire, such as the northeastern Alps, but also areas beyond its borders, such as the mountainous regions of the Carpathians or the Greater Caucasus. The incremental C and N stable isotope analysis provided detailed information into Offord Cluny 203645’s complex life history of long-distance migration, revealing two moments of dietary change: first at ~5 years of age, from a predominately C_4_ to a mixed C_3_/C_4_ diet, and then again at ~9 years of age to a diet based predominantly on C_3_ resources, possibly reflecting two episodes of migration (Figure 3). Linear defects, or enamel hypoplasia, on the crowns of nine teeth from Offord Cluny 203645 may reflect periods of arrested growth during episodes of malnutrition or illness.^33,34^ The location of these defects suggests they occurred around the age of 5 years, overlapping with the timing of the first observed shift in diet, and might therefore reflect physiological stress associated with dietary changes and possible migration. The two shifts in diet might reflect a hiatus in his journey westwards before reaching Britain and would be consistent with a period of time spent in central or southeastern Europe. The δ^13^C value corresponding to ~13 years of age is closer to (but still slightly more elevated than) the values typically observed in Roman Britain,^35,36^ and thus it is possible that he only moved to Britain later in his life.

The impact of (possibly transient) long-distance individual mobility and admixture at urban sites during historical periods^37,38^ has been recently highlighted across a variety of sites in Europe, North Africa, the Caucasus, and the Levant.^12^ In Britain, in addition to one outlier individual with ancestry related to present-day Near Eastern populations in the possible military or gladiator cemetery at Driffield Terrace, in present-day York (*Eboracum*, a major urban center and provincial capital),^9^ isotope signatures consistent with continental Europe and the Mediterranean basin have also been reported at other important urban Roman settlements^36,39,40^. By contrast, Offord Cluny 203645 was found in what would have been a rural location, albeit within a substantial farmstead that later developed into a villa complex. The skeleton was not recovered from one of the small formal Romano-British cemeteries found along the modern A14 road, but from an isolated burial that had been placed within a former trackway ditch toward the fringes of the farmstead. Isolated burials outside of formal cemeteries in peripheral unfurnished graves are a common feature of early-mid Roman farmsteads and villas.^41,42^ It is usually unclear who was placed in these isolated burials, though the very act of interment itself does distinguish them, with the majority of the rural population during the early-mid Roman period having been subjected to funerary rites which left little archaeological trace (e.g., excarnation).

Contributions of Caucasus- or Pontic-Caspian-associated ancestry, usually admixed with local populations, have been identified in Roman cemeteries in other parts of the empire, such as in Italy or the Balkans^4,5^ (Figure 1C). The second century CE witnessed a series of interactions between the Roman Empire and the inhabitants of the Caucasus, including a brief period between 114 and 117 CE when Greater Armenia became a Roman province,^43^ as well as several documented Sarmatian-Alan incursions into the Roman-controlled South Caucasus.^2^ In the northeastern fringes of the empire, the Marcomannic Wars (166–180 CE) pitted the Romans against Germanic and Sarmatian peoples.^1^ All of these events could have promoted long-distance mobility of groups or individuals carrying Caucasus- and Sarmatian-related ancestry into and within the Roman Empire.

The age at death (18–25 years old) and history of migration (based both on genetic ancestry and stable isotope evidence) we have obtained from Offord Cluny 203645 could be consistent with this individual having come to Britain as part of a military movement, either as part of a soldier’s family or as a soldier himself. One possibility, given the radiocarbon date obtained (126–228 cal. CE; median 176 cal. CE), would be the historically attested deployment of Sarmatian cavalry in 175 CE, following Roman emperor Marcus Aurelius’s victory in the Marcomannic Wars, as described by the Roman historian Cassius Dio.^4,5^ In this scenario, the dietary shifts we see in Offord Cluny 203645 would be explicable if he was associated with groups of Sarmatians who moved into central Europe before or during the Marcomannic wars,^3^ although the plausibility of this interpretation depends on whether children were likely to have been part of movements of Sarmatians across Europe. Little is known about where the 5,500 Sarmatians were stationed in Britain. There are suggestions of Sarmatian horse equipment from Chesters on Hadrian’s Wall and epigraphic evidence for them from Ribchester, *Bremetennacum Veteranorum* in northwest England and Catterick, *Cataractonium* in northeast England,^4,44^ all a considerable distance from the A14 sites in Cambridgeshire.

Other interpretations that could plausibly account for long-distance movement across the Roman Empire include, although are not limited to, governance of the empire, economic migration, or slavery. The absence of grave goods and the generally unremarkable nature of his grave prevents assessment of which scenario is most likely. A plausible explanation is that he died while en route somewhere, although this scenario may be weakened by the location of his burial one kilometer to the west of a major Roman road connecting Sandy and Godmanchester, *Durovigutum* (Figure 1A). An alternative hypothesis is that Offord Cluny 203645 was associated with the farmstead, possibly integrated within a rural civilian community.

Whatever the reasons for the journeys Offord Cluny 203645 took over his short lifetime, his burial highlights the impact that the Roman Empire had on rural locations in Britain (and probably elsewhere) in terms of increasing long-distance mobility and introducing genetic ancestry from the far fringes or even regions outside of the Roman Empire. Future identification of additional individuals with Caucasus- and/or Sarmatian-related ancestry in Roman Britain, particularly examples accompanied by grave goods or from indicative contexts (e.g., military), will offer more insights into how people who carried these ancestries arrived in Britain.

## Star⋆Methods

Detailed methods are provided in the online version of this paper and include the following:

- KEY RESOURCES TABLE
- RESOURCE AVAILABILITY
  ○ Lead contact
  ○ Materials availability
  ○ Data and code availability
- EXPERIMENTAL MODEL AND STUDY PARTICIPANT DETAILS
  ○ Archaeological context
  ○ Skeletal samples
- METHOD DETAILS
  ○ DNA sampling and sequencing
  ○ Strontium isotopes
  ○ Oxygen isotopes
  ○ Carbon and nitrogen isotopes
- QUANTIFICATION AND STATISTICAL ANALYSIS
  ○ Sequencing data processing and aDNA authentication
  ○ Genotyping and compiled datasets
  ○ Population analyses

## Star⋆Methods

### Key Resources Table

**Table:** 

REAGENT or RESOURCE	SOURCE	IDENTIFIER
Biological samples		
Archeological samples: right temporal bone; second right mandibular molar; second right maxillary molar	This study	Skeleton (Sk) 203645 (Additional identifiers: Burial 20.507; C10271)
Chemicals, peptides, and recombinant proteins		
T4 DNA Ligase (5 U/μL)	Fisher Scientific	Cat# EL0012
FastAP Thermosensitive Alkaline Phosphatase (1 U/μL)	Fisher Scientific	Cat# EF0651
Klenow Fragment (10U/ul)	Fisher Scientific	Cat# EP0052
T4 Polynucleotide Kinase (10 U/μL)	Fisher Scientific	Cat# EK0031
T4 RNA Ligase Reaction Buffer	NEB	Cat# B0216
ATP Solution (100 mM)	Fisher Scientific	Cat# R0441
dNTP Mix (25 mM each)	VWR	Cat# 733-1854
Dynabeads MyOne Streptavidin C1 beads	Thermo Fisher Scientific	Cat# 65002
G-Biosciences Silica Magnetic Beads	VWR	Cat# 786-915
AccuPrime *Pfx* DNA Polymerase	Thermo Fisher Scientific	Cat# 12344024
Sera-Mag SpeedBeads, magnetic carboxylate-modified microparticles	Sigma-Aldrich	Cat# GE65152105050250
Herculase II Fusion DNA Polymerase	Agilent	Cat# 600679
pUC19 vector	NEB	Cat# N3041S
Hydrochloric acid, >37% (0.5 M Solution)	Sigma-Aldrich	Cat#30721-M
6 M Hydrochloric acid	Romil	Distilled and titrated in-house
3 M Nitric acid	Romil	Distilled and titrated in-house
Sr-Spec Resin	Triskem	Cat# SR-B25-S
Critical commercial assays		
MinElute PCR Purification Kit	Qiagen	Cat# 28004
High Pure Viral Nucleic Acid Large Volume Kit	Roche	Cat# 05114403001
Maxima Probe qPCR Master Mix	Fisher Scientific	Cat# K0262
Agilent DNA 1000 Kit	Agilent	Cat# 5067-1504
Deposited data		
Offord Cluny Sk203645 (Burial 20.507, C10271): FASTQ files and mapped BAM file	This study	https://www.ebi.ac.uk/ena/browser/view/PRJEB67353
Human reference genome NCBI build 37, GRCh37	Genome Reference Consortium	https://www.ncbi.nlm.nih.gov/grc/human
Comparison shotgun data	Allentoft et al.^45^	https://www.ebi.ac.uk/ena/browser/view/PRJEB9021
Comparison shotgun data	Yaka et al.^46^	https://www.ebi.ac.uk/ena/browser/view/PRJEB39316
Comparison shotgun data	Hofmanova et al.^47^	https://www.ebi.ac.uk/ena/browser/view/PRJEB11848
Comparison shotgun data	Omrak et al.^48^	https://www.ebi.ac.uk/ena/browser/view/PRJEB12155
Comparison shotgun data	Antonio et al.^12^	https://www.ebi.ac.uk/ena/browser/view/PRJEB53564
Comparison shotgun data	Gamba et al.^15^	https://www.ebi.ac.uk/ena/browser/view/PRJNA240906
Comparison shotgun data	Marchi et al.^49^	https://www.ebi.ac.uk/ena/browser/view/PRJEB50857
Comparison shotgun data	Jones et al.^50^	https://www.ebi.ac.uk/ena/browser/view/PRJEB11364
Comparison shotgun data	Saag et al.^51^	https://www.ebi.ac.uk/ena/browser/view/PRJEB40698
Comparison shotgun data	Fu et al.^52^	https://www.ebi.ac.uk/ena/browser/view/PRJEB13123
Comparison shotgun data	de Barros Damgaard et al.^53^	https://www.ebi.ac.uk/ena/browser/view/PRJEB26349
Comparison shotgun data	Schiffels et al.^20^	https://www.ebi.ac.uk/ena/browser/view/PRJEB6915
Comparison shotgun data	Martiniano et al.^9^	https://www.ebi.ac.uk/ena/browser/view/PRJEB11004
Comparison shotgun data	Dulias et al.^54^	https://www.ebi.ac.uk/ena/browser/view/PRJEB46830
Comparison shotgun data	Gonzalez-Fortes et al.^55^	https://www.ebi.ac.uk/ena/browser/view/PRJEB20616
Comparison shotgun data	Antonio et al.^38^	https://www.ebi.ac.uk/ena/browser/view/PRJEB32566
Comparison shotgun data	de Barros Damgaard et al.^10^	https://www.ebi.ac.uk/ena/browser/view/PRJEB20658
Comparison shotgun data	Krzewinska et al.^56^	https://www.ebi.ac.uk/ena/browser/view/PRJEB27628
Comparison shotgun data	Schlebusch et al.^57^	https://www.ebi.ac.uk/ena/browser/view/PRJEB22660
Comparison shotgun data	Lazaridis et al.^58^	https://www.ebi.ac.uk/ena/browser/view/PRJEB6272
Comparison shotgun data	Olalde et al.^59^	https://www.ebi.ac.uk/ena/browser/view/PRJNA230689
Comparison shotgun data	Brace et al.^18^	https://www.ebi.ac.uk/ena/browser/view/PRJEB31249
Comparison shotgun data	Sikora et al.^60^	https://www.ebi.ac.uk/ena/browser/view/PRJEB29700
“Allen Ancient DNA Resource” v.54	Mallick et al.^61^	https://dataverse.harvard.edu/dataset.xhtml? persistentId=doi:10.7910/DVN/FFIDCW
1000 Genomes Project (1KGP) phase 3	The 1000 Genomes Project Consortium^62^	https://www.internationalgenome.org/category/phase-3/
YFull YTree v.11.01.00	N/A	https://www.yfull.com/tree/
ISOGG Y-DNA Haplogroup Tree 2019-2020	N/A	https://isogg.org/tree/
PhyloTree v.17	van Oven^63^	https://www.phylotree.org/index.htm
Oligonucleotides		
ssDNA library preparation oligonucleotides	Gansauge et al.^64^; Sigma-Aldrich	N/A
CL304, positive control template	Gansauge et al.^64^; Sigma-Aldrich	N/A
P5 and P7 index primers	Gansauge and Meyer^65^; Sigma-Aldrich	N/A
IS5/IS5 biotinylated and IS6, forward and reverse primers	Gansauge et al.^64^; Sigma-Aldrich	N/A
qPCR standard, forward and reverse primers and qPCR probes	Gansauge et al.^64^; Sigma-Aldrich	N/A
forward and reverse primers for preparing gel markers	Gansauge et al.^64^; Sigma-Aldrich	N/A
CL72, sequencing read 1 primer for ssDNA libraries	Gansauge et al.^64^; Sigma-Aldrich	N/A
Software and algorithms		
nf-core/eager v.2.3.3	Fellows Yates et al.^66^	https://nf-co.re/eager/2.3.3
fastp v.0.20.1	Chen et al.^67^	https://github.com/OpenGene/fastp
AdapterRemoval v2.3.1	Schubert et al.^68^	https://github.com/MikkelSchubert/adapterremoval
bwa v.0.7.17-r1188	Li and Durbin^69^	https://github.com/lh3/bwa/releases/tag/v0.7.17
Dedup v.0.12.8	Peltzer et al.^70^	https://github.com/apeltzer/DeDup/releases/tag/0.12.8
*ry_compute.py*	Skoglund et al.^6^	https://github.com/pontussk/ry_compute
ANGSD v.0.933	Korneliussen et al.^71^	http://www.popgen.dk/angsd/index.php/ANGSD
schmutzi v.1.5.6	Renaud et al.^72^	https://github.com/grenaud/schmutzi
DamageProfiler v.1.1	Neukamm et al.^73^	https://github.com/Integrative-Transcriptomics/DamageProfiler
Yleaf v.3.1	Ralf et al.^74^	https://github.com/genid/Yleaf
samtools v.1.3.1	Li et al.^75^	https://www.htslib.org/download/
Haplogrep2	Weissensteiner et al.^76^	https://haplogrep.i-med.ac.at/haplogrep2
sequenceTools v.1.5.2	N/A	https://github.com/stschiff/sequenceTools
PLINK v.1.9	Purcell et al.^77^	https://www.cog-genomics.org/plink/
EIGENSOFT v.6.1.4	Patterson et al.^78^	https://github.com/DReichLab/EIG
ADMIXTOOLS v.5.0	Patterson et al.^79^	https://github.com/DReichLab/AdmixTools
*qpAdm_wrapper.py*	N/A	https://github.com/pontussk/qpAdm_wrapper
POPSTATS	Skoglund et al.^80^	https://github.com/pontussk/popstats

### Resource Availability

#### Lead contact

Further information and requests for resources should be directed to and will be fulfilled by the lead contact, Pontus Skoglund (pontus.skoglund@crick.ac.uk).

#### Materials availability

This study did not generate new unique reagents.

### Experimental Model and Study Participant Details

#### Archaeological context

Between 2016 and 2019 MOLA Headland Infrastructure excavated a series of multiperiod sites in Cambridgeshire, eastern England on behalf of National Highways as part of the A14 Cambridge-Huntingdon improvement scheme. Amongst other features, these excavations provided evidence of a well-populated rural Roman landscape comprising a series of complex farmsteads, associated small cemeteries, villa sites, extensive field systems and isolated human burials. Here, we present genetic and isotopic evidence of an outlier individual whose remains were recovered from a farmstead (Settlement 2 within the River Great Ouse Landscape Block of excavations) on the floodplain and gravel terrace of the River Great Ouse, north of the village of Offord Cluny during the A14 excavations (Figure 1A).

The skeletal remains were recovered from an isolated inhumation. The body appeared to have been laid carefully, slightly flexed on its left side in a north-south orientation with the head to the south and with the hands crossed in front of the upper legs (Figure S1A). While there was no evidence of a wrapping or shroud, there may have been some constriction of the body, particularly at the hands and the knees. The proximity of the hands suggests they may have been deliberately placed, but it is not possible to say whether they were wrapped or bound. Post-depositional movement of the upper limbs, probably caused by slumping within the grave, has caused some loss of articulation in the area of the right wrist which may both reflect and mask the original position of the right wrist and hand. There was no detectable grave cut and no grave goods, although any perishable items would not have survived.

#### Skeletal samples

Skeleton 203645 (Burial 20.507; Crick ancient genomics lab ID: C10271) comprised the remains of a young adult (aged 18–25 years). Age estimation was based on observations of dental development and epiphyseal fusion.^81,82^ The bone was moderately-well preserved but the spine, pelvis and lower limbs were degraded and fragmented, which prevented estimation of sex from dimorphic features of the skull and pelvis. Linear enamel hypoplastic defects were observed in nine teeth, probably occurring around the age of ~5 years based on their location.

We collected the right petrous temporal bone from this individual for aDNA analysis, and the second right mandibular molar tooth for stable isotope analysis. In addition, the second right maxillary molar was radiocarbon dated to 1867 ± 16 BP (SUERC-105720 (GU61561)) at the Scottish Universities Environmental Research Centre AMS Laboratory, corresponding to 126–228 cal. CE (95.4% probability) after calibration with OxCal v4.4^7^ using IntCal20^8^ (Figure 1B). Minimally-destructive sampling for aDNA analysis followed guidelines issued by the Department for Culture, Media and Sport (DCMS) and the Advisory Panel on the Archaeology of Burials in England (APABE) (apabe.archaeologyuk.org).

### Method Details

#### DNA sampling and sequencing

DNA sampling and pre-amplification protocols were performed in specialized clean rooms at the Francis Crick Institute. We drilled multiple subsamples of fine bone powder from the cochlear portion of the petrous bone^83^ using a Emax EVOlution (EV410) micromotor system with disposable carbide round burs.

We extracted DNA from a subsample of 18.60 mg of bone powder (using 700 μL of lysis buffer),^84^ and prepared double-indexed single-stranded (ss) DNA libraries^64,85^ without performing any UDG-treatment, using automated liquid-handling systems (Agilent Bravo Workstations). We included negative extraction and library controls to rule out contamination arising during lab procedures. Libraries (including negative controls) were initially screened in an Illumina HiSeq 4000 instrument, resulting in ~2.6M paired-end (PE) reads of 100 bp. Following assessment of DNA preservation, we re-sequenced the library twice on the Illumina NovaSeq S4 platform, for a total of ~1.9 billion PE reads, using PE sequencing for 100 cycles (for one of the sequencing rounds we subjected the library to a gel-excision protocol^64^ to remove DNA sequences <35 bp and >150 bp).

#### Strontium isotopes

Core enamel samples (~5 mg) were prepared for strontium (Sr) isotope analysis using column chemistry methods^86^ at the Arthur Holmes Isotope Geology Laboratory (AHIGL), Durham University. Samples were digested overnight in 3M HNO_3_ on a hotplate at 100° C before being loaded onto cleaned and preconditioned columns containing Eichrom strontium-specific resin. A purified Sr fraction was eluted from the column in 400 μL H_2_O and acidified with 15.5M HNO_3_ to yield a 3% HNO_3_ solution. Samples were aspirated using an ESI PFA-50 nebulizer coupled to a Glass Expansion Cinnabar micro-cyclonic spraychamber. Sr isotopes were measured using a static multi-collection routine with each measurement comprising a single block of 50 cycles with and integration time of 4s per cycle (total analysis time ~3.5 mins). Instrumental mass bias was corrected for using an ^88^Sr/^86^Sr ratio of 8.375209 (the reciprocal of the more commonly used ^86^Sr/^88^Sr ratio of 0.1194) and an exponential law. Corrections for isobaric interferences from Rb and Kr on ^87^Sr and ^86^Sr were performed using ^85^Rb and ^83^Kr as the monitor masses but were insignificant. In all samples the ^85^Rb intensity was < 1mV with an ^85^Rb/^86^Sr ratio of < 0.0003 (average 0.0001). ^83^Kr was between 0.32 and 0.39mV in all samples. Samples were measured during a single analytical session during which the average ^87^Sr/^86^Sr ratio and reproducibility for the international isotope reference material NBS987 was 0.710269 ± 0.000013 (2σ; n = 12). Maximum error based on internal precision of individual analysis and analytical reproducibility of the reference material is 0.000013 (2σ). Sr isotope data for samples is normalized to an ‘accepted’ value for NBS987 of 0.71024.

#### Oxygen isotopes

Core enamel samples (~15 mg) were transferred to Iso Analytical for stable isotope analysis where samples were weighed into Exetainer tubes and flushed with 99.995% helium. Carbonate in the samples was converted to CO_2_ by adding phosphoric acid and letting the samples sit overnight for the reaction to occur. Reference materials (IA-R022, NBS-18, and IA-R066) were prepared along the same methods. CO_2_ from the samples was then analyzed by Continuous Flow-Isotope Ratio Mass Spectrometry (CF-IRMS). The CO_2_ was sampled from the Exetainer tubes into a continuously flowing He stream using a double holed needle. The CO_2_ was resolved on a packed column gas chromatograph and the resultant chromatographic peak carried forward into the ion source of a Europa Scientific 20-20 IRMS where it was ionized and accelerated. Gas species of different mass were separated in a magnetic field then simultaneously measured using a Faraday cup collector array to measure the isotopomers of CO_2_ at m/z 44, 45, and 46. The phosphoric acid used for digestion was prepared in accordance with Coplen et al. (1983)^87^ and was injected through the septum into the vials. 20% of samples were run in duplicate.

#### Carbon and nitrogen isotopes

A dentine sample was collected from the root of a second molar and collagen extracted for incremental carbon and nitrogen isotope analysis following the Beaumont et al. (2014)^88^ method. Each increment within the dietary profile constitutes a running average (rather than a discrete snapshot of diet) due to the orientation of the dentine incremental layers and how many are included in each increment. In human molar teeth, the orientation of these layers is relatively horizontal in the tooth crown (increments 1–6) and becomes more vertical in the tooth root (increments 7–15), suggesting temporal resolution may be higher in the crown than in the root. For the second molar the peak velocity is likely to be during increments 1–2 within the crown and increments 7–9 within the root.^31,32^

Extracted collagen was weighed into tin capsules and measured in duplicate using a Thermo Scientific Delta V Advantage isotope ratio mass spectrometer in the Stable Isotope Biogeochemistry Laboratory (SIBL), Durham University. Calibration using internal reference samples (e.g., Glutamic Acid, Glycine, SPAR and Urea) and international reference standards (e.g., USGS 24, USGS 40, IAEA 600, IAEA N1, IAEA N2) determined a standard deviation of ±0.1‰ (1σ) for collagen carbon and nitrogen isotopes. Replicate analysis of collagen samples averaged a standard deviation of ±0.2‰ (1σ).

### Quantification and Statistical Analysis

#### Sequencing data processing and aDNA authentication

Sequencing data were processed using nf-core/eager^66^ v.2.3.3. We preprocessed PE sequencing reads with fastp^67^ v.0.20.1, followed by PE merging and filtering for minimum read length of 35 bp with AdapterRemoval^68^ v2.3.1 (*--collapse, --preserve5p, --trimns, --trimqualities, --minlength 35, --minquality 20, --minadapteroverlap 1*). For libraries sequenced on the Illumina NovaSeq S4 platform, we performed lane merging before mapping to the human reference genome (hs37d5) using bwa^69^ v.0.7.17-r1188 *aln* (*-n 0.01, -l 1024, -k 2*) and *samse*. We removed PCR duplicates with Dedup^70^ v.0.12.8. To generate files containing only mitochondrial DNA (mtDNA) reads, we realigned mapped human reads to rCRS (GenBank: NC_012920).

We assigned the libraries as karyotypically male (XY).^6^ We estimated contamination on the X-chromosome using ANGSD^71^ v.0.933, and assessed mtDNA contamination using schmutzi^72^ v.1.5.6 (*contDeam.pl --library single*) (Data S1A). We merged BAM files using samtools^75^ v.1.3.1 *merge*, and subsequently removed duplicates (Dedup *-m*), resulting in a final average nuclear coverage of ~5.4x (~4.12x after filtering for mapping quality (MQ) > 30). We used DamageProfiler^73^ v.1.1 (*-sslib*) to assess 5′- and 3′-end C>T substitutions (Figure S1B).

We classified the Y-chromosome lineage using Yleaf^74^ v.3.1 (*-r3, -q30, -dh, -hc*) and cross-checked against YFull YTree v.11.01.00 (https://www.yfull.com/tree/) and ISOGG Y-DNA Haplogroup Tree 2019–2020 (https://isogg.org/tree/). For mtDNA haplogroup classification we used Haplogrep2^76^ based on PhyloTree^63^ v.17, restricting the data to sites covered by at least four sequencing reads with MQ > 30 and base quality >30, and allele frequency >0.90.

#### Genotyping and compiled datasets

We used samtools *mpileup* (*-R, -B, -q30, -Q30*) and pileupCaller with the options *--randomHaploid* and *--singleStrandMode* (sequenceTools v.1.5.2; https://github.com/stschiff/sequenceTools) to call pseudo-haploid autosomal SNPs overlapping with with the ‘1240k’ panel^89^ and with ~3,868,200 biallelic transversions with 1% minor allele frequency (maf) on the 1000 Genomes Project (1KGP) phase 3 global panel,^62^ hereafter referred to as ‘1KGP transversion sites’ (SNP list was generated using PLINK v.1.9^77^ *--biallelic-only strict, --maf 0.01*).

We extracted genotypes reported in the ‘Allen Ancient DNA Resource’^61^ v.54 (https://doi.org/10.7910/DVN/FFIDCW). We selected individuals from England dating to the Iron Age and Roman period, individuals with latitude values between 30 and 64, longitude between −20 and 60 and mean date between 2000–1475 BP (as reported in the ‘Allen Ancient DNA Resource’ v.54 dataset), but excluding individuals from early mediaeval contexts. Following a preliminary PCA analysis, we also selected individuals associated with Sarmatian contexts and individuals from the Caucasus dating to the Late Bronze Age and Iron Age. We retained only unrelated individuals with >35,000 SNPs overlapping with the ‘1240k’ panel and >20,000 SNPs overlapping with the Affymetrix Human Origins (HO) array and with no evidence of contamination. We removed close relatives by keeping the individual with the highest number of genotyped SNPs. The final ‘1240k dataset’ comprised 677 previously reported individuals.^9,10,12,13,20,38,45,54,56,89,90–104^

We compiled an additional dataset comprising 128 published individuals with whole-genome shotgun data available that we genotyped with samtools *mpileup* and pileupCaller *--randomHaploid* using the ‘1KGP transversion sites’ list, as described above (Data S2D). This comprised a subset of the individuals included in the ‘1240k dataset’ plus additional outgroup and reference populations^15,45,46–53,55,57–60^ and was used for all population analyses except PCA.

#### Population analyses

We used smartpca with options *shrinkmode: YES* and *lsqproject: YES* (EIGENSOFT^78^ v.6.1.4) to project Offord Cluny 203645 alongside 677 previously published ancient individuals (‘1240k dataset’) on Principal Components (PCs) computed using ~600k SNPs from the HO array genotyped in 1388 present-day individuals from Europe, the Near East and the Caucasus^14,58,79^ (Data S2E).

We first ran *qpAdm* framework using a wrapper based on ADMIXTOOLS^79^ v.5.0 (https://github.com/pontussk/qpAdm_wrapper), adapting a model optimized for post-Bronze Age Britain,^90^ with a fixed set of outgroups (ancient sub-Saharan African individuals (*South_Africa_400BP, n* = 4), individuals genetically similar to Iron Gates Mesolithic Hunter-gatherers (*n* = 3), Anatolia Neolithic individuals (*Anatolia_N, n* = 18), and Afanasievo individuals (*n* = 4)) and three distal sources: Western European Hunter-Gatherers (*WHG, n* = 7), Neolithic individuals from southeast Europe (*Balkan_N*, representing European Early Farmers (EEFs) ancestry, *n* = 9) and Yamnaya individuals (representing Steppe-associated ancestry, *n* = 7) (Data S2F). This analysis showed that Offord Cluny 203645 did not harbor *WHG*-related ancestry (p = 1.65E−10) that is otherwise present in the majority of sampled individuals from post-Bronze Age Western and Central Europe,^17,90^ and observed in proportions ranging from 15.0 to 21.5% in all non-outlier individuals from the Driffield Terrace cemetery (Figure S2B and Data S2F). Following this result, we then tested other distal 2-source models (*--sources 2*), using a rotating approach^105^ through a list of reference populations comprising the outgroups and sources in the previous model plus Caucasus Hunter-Gatherers (*CHG, n* = 2) and Eastern European Hunter-gatherers (*EHG, n* = 3) (Figure S2C and Data S2G).

To find more proximal sources of ancestry, we tested different *qpWave* (*--qpwave –sources 1*) and *qpAdm* (*--sources 2*) models using a rotating approach on a selection of West Eurasian populations and additional outgroups (for a total of 4 different reference lists): *South_Africa_400BP* (*n* = 4), *Yana_UP* (*n* = 2), *Lithuania_Marvele* (*n* = 4), *Portugal_LateRoman* (*n* = 5), *Italy_ImperialRoman* (*n* = 20), *England_IA* (*n* = 5) or *England_Roman* (*n* = 6), *Russia_Sarmatian_PonticSteppe* (*n* = 7), *Russia_Sarmatian_SouthernUrals* (*n* = 4), *Russia_Sarmatian_Alan* (*n* = 5), *Armenia_LBA* (*n* = 7), *Armenia_Antiquity* (*n* = 6). We confirmed that none of the Sarmatian groups formed a clade with each other (Data S2B). *Armenia_LBA* was excluded when testing more temporally proximal models. All tested models with different reference lists are shown in Data S2B and S2C.

We ran *f*4-statistics using POPSTATS^80^ (*--f4, --haploidize, --informative*) to untangle patterns of shared genetic drift amongst ancient individuals from Roman Britain (Offord Cluny 203645, and previously published individuals from Driffield Terrace^9^), different ancient populations with connections to the Caucasus or the Pontic-Caspian region (*Armenia_LBA, Armenia_Antiquity, Russia_Sarmatian_Alan*, and *Russia_Sarmatian_PonticSteppe*), and *England_IA*.

## Supplementary Material

Supplemental information can be found online at https://doi.org/10.1016/j.cub.2023.11.049.

Data S1

Data S2

Data S3

Figure S1

## Data Availability

Sequencing data (FASTQ and BAM files) are available on ENA: PRJEB67353. This paper does not report original code. Any additional information required to reanalyze the data reported in this paper is available from the lead contact upon request.

## Highlights

- Ancestry outlier identified in rural Roman Britain dating to 126–228 cal. CE
- Genetically related to contemporary Sarmatian- and Caucasus-associated groups
- Stable isotope analysis reveals life history of mobility
- Deployment of Sarmatian cavalry to Britain in 175 CE is a plausible explanation

## In brief

Silva et al. identify a Roman-era individual buried in rural Britain with ancestry related to the Caucasus- and Sarmatian-associated groups. Isotope analysis evidences long-range childhood mobility. These results may be linked to historical movements of Sarmatians and highlight long-distance mobility reaching rural regions of the Roman Empire.
