# Supplementary material for: An individual with Sarmatian-related ancestry in Roman Britain
Source: Curr Biol. Author manuscript; Available in PMC 2025 Jun 24. (PMC7617811; doi:10.1016/j.cub.2023.11.049)
Supplement: Figure S1 [file EMS206332-supplement-Figure_S1.pdf]

Current Biology, Volume *34*

Supplemental Information

An individual with Sarmatian-related ancestry in Roman Britain

Marina Silva, Thomas Booth, Joanna Moore, Kyriaki Anastasiadou, Don Walker, Alexandre Gilardet, Christopher Barrington, Monica Kelly, Mia Williams, Michael Henderson, Alex Smith, David Bowsher, Janet Montgomery, and Pontus Skoglund

A

B

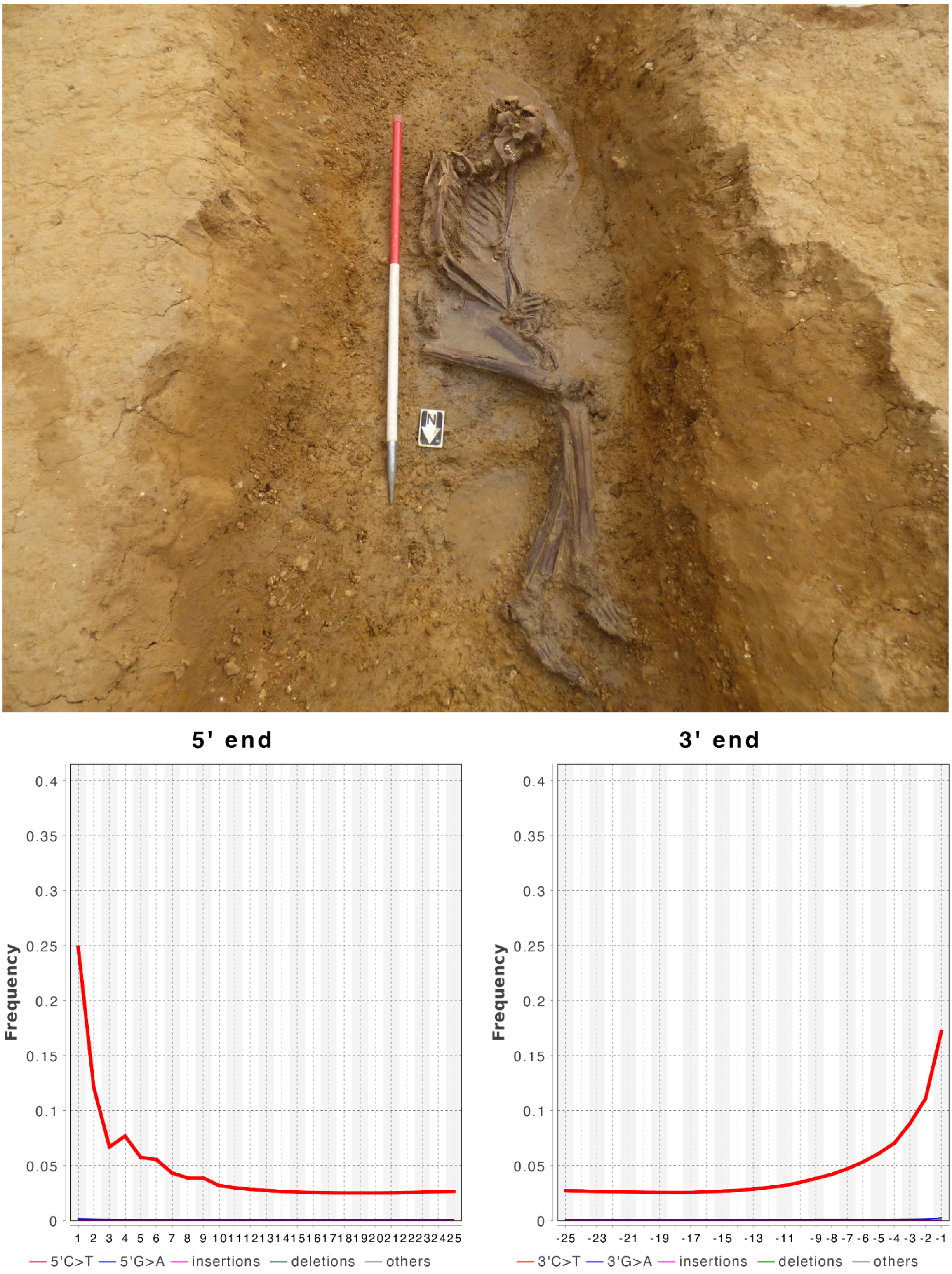

Figure S1. Skeletal remains and aDNA authenticity, *related to Figure 1*.

A) Offord Cluny 203645/Burial 20.507 inhumation (Crick ancient genomics lab ID: C10271). **B)** Frequency of misincorporations at the 5'- (left) and 3'- (right) ends of sequencing reads in the form of C>T, denoting postmortem molecular damage (plot generated using DamageProfiler). Sequencing metrics shown in Data S1.

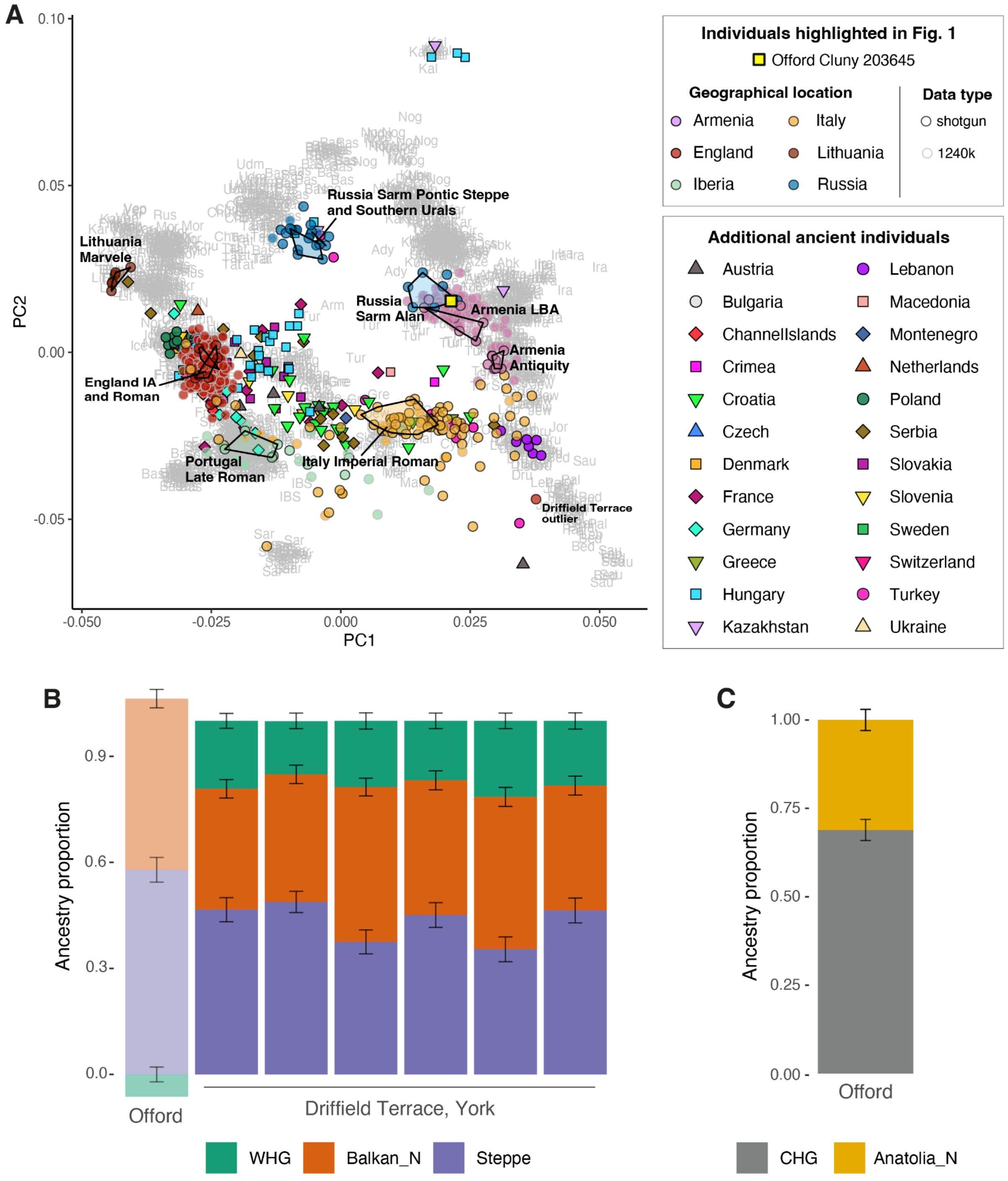

Figure S2. Ancestry outlier Offord Cluny 203645, *related to Figures 1 and 2.*
A) Principal Component Analysis (PCA) shown in Figure 1C, with additional projected ancient individuals (dated to between 2000-1475 BP) coloured according to geography. Offord Cluny 203645 is represented by a yellow square. Individuals included in the populations used as sources in the *qpWave/qpAdm* models are grouped (as highlighted in Figure 1), with additional individuals from the same regions coloured according to geographical location and data type (whole-genome shotgun sequencing or '1240k' SNP capture). Present day individuals are indicated by the first 3 letters of their population label, as reported in Data S2E. **B)** Fixed *qpAdm* distal model using 3 sources: Western European Hunter-gatherers (*WHG*), Balkan Neolithic (*Balkan_N*) and Steppe (Data S2F). Transparency denotes the rejected model (p<0.05). **C)** 2-source *qpAdm* distal model with highest p-value (p=0.551): Caucasus Hunter-gatherers (*CHG*) and Anatolia Neolithic (*Anatolia_N*) (Data S2G). Individuals included in the models and population grouping listed in Data S2D.

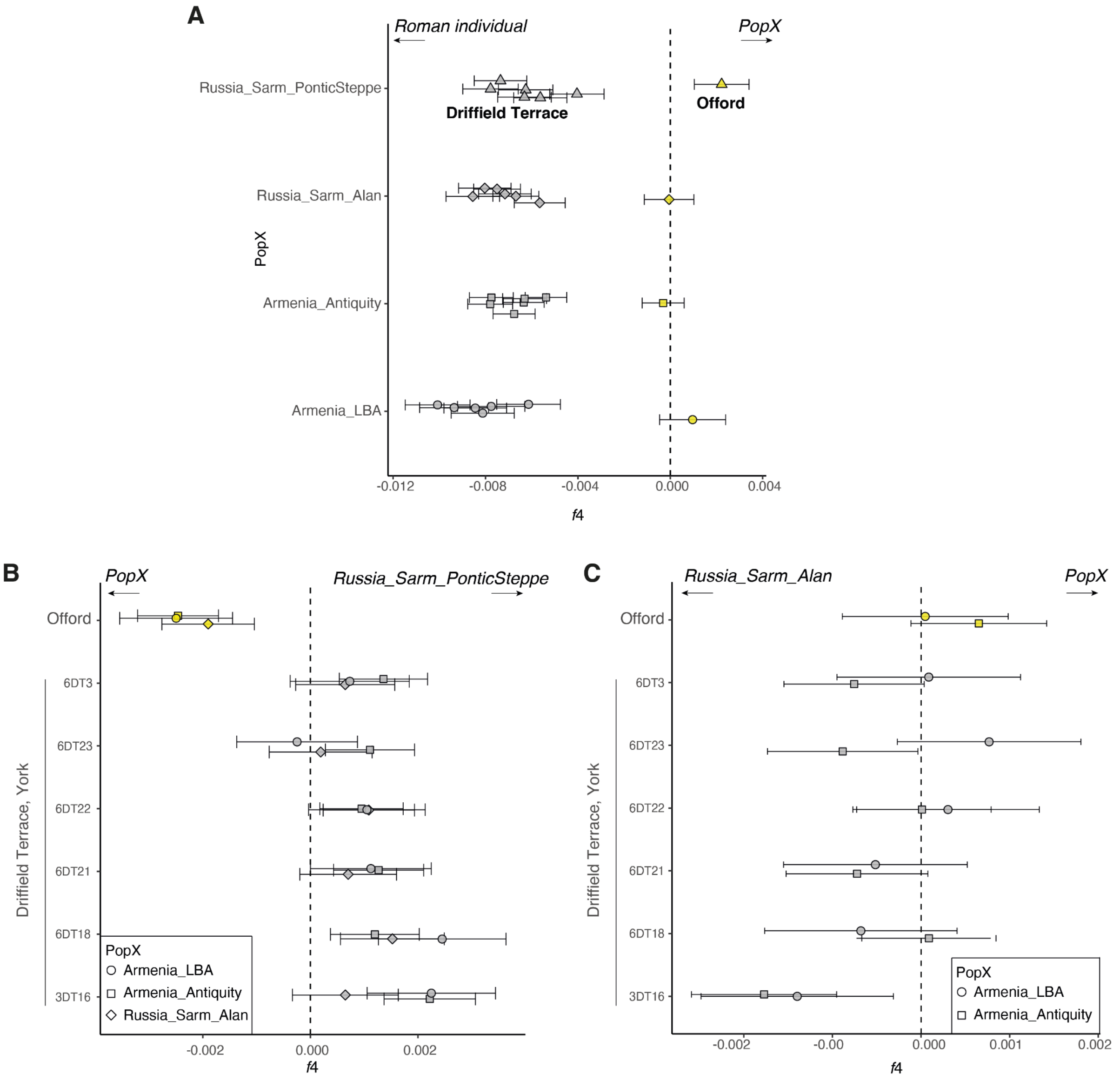

Figure S3. f_4-statistics, *related to Figure 1*.
Data points referring to Offord Cluny 203645 are shown in yellow, and data points for individuals from Driffield Terrace site in York shown in grey. Error bars denote 1 standard error. See also Data S2A. **A)** *f_4(South_Africa_400BP, England_IA; Roman individual, PopX)*. **B)** *f_4(South_Africa_400BP, Roman individual; PopX, Russia_Sarmatian_PonticSteppe)*. **C)** *f_4(South_Africa_400BP, Roman individual; Russia_Sarmatian_Alan, PopX).*
